# Effect of the MiR-99b and MiR-135b on peritoneal carcinomatosis and liver metastasis in colorectal cancer

**DOI:** 10.1016/j.clinsp.2023.100271

**Published:** 2023-08-26

**Authors:** Mehmet Aziret, Gamze Eskiler, Gözde Çakırsoy Çakar, Metin Ercan, Cemil Bilir, Erdal Polat, Havva Belma Koçer, Ebru Kayra Yıldırım, Mustafa Duman, Asuman Deveci Özkan

**Affiliations:** a University of Health Science, Balıkesir City Training and Research Hospital, Balıkesir, Turkey; b Sakarya Training and Research Hospital, Sakarya, Turkey; c Istinye University, Istanbul, Turkey; d University of Health Science, Kartal Kosuyolu Training and Research Hospital, Istanbul, Turkey

**Keywords:** Colorectal cancer, Liver metastasis, MiR-99b, MiR-135b, Peritonitis carcinomatosis

## Abstract

**Aim:**

This study aimed to evaluate the expression levels of miR-99b and miR-135b in peritoneal carcinoma and liver metastases associated with Colorectal Cancer (CRC), assess their association with the intracellular signaling pathway proteins Kirsten Rat Sarcoma Virus (KRAS) and Akt, and investigate their effects on survival.

**Materials and methods:**

Changes in the KRAS gene and Akt proteins, expression levels of miR-99b and miR-135b, and factors affecting survival were compared between colorectal cancer-associated peritoneal carcinomatosis and liver metastasis.

**Results:**

The expression levels of miR-99b and miR-135b and the immunohistochemical grade classification score of Akt were higher in colorectal cancer, peritoneal carcinomatosis, and liver metastasis than in normal tissues (*p* < 0.05). MiR-99b expression was highest in CRC, whereas miR-135b expression was highest in peritoneal carcinomatosis (*p* < 0.05). The expression level of miR-99b decreased and that of miR-135b increased in peritoneal and liver metastases compared with that in the tumor tissue. MiR-99b, Akt, and recurrence were risk factors that affected the overall survival rate in the model of clinical predictions (*p* = 0.045, *p* = 0.006, and *p* = 0.012, respectively).

**Conclusion:**

While the expression of miR-99b was highest in the primary tumor, its decrease in liver metastasis and peritoneal carcinomatosis suggests that miR-99b has a protective effect against liver metastasis and peritoneal carcinomatosis. However, the detection of miR-135b expression was highest in peritoneal carcinomatosis and liver metastasis compared with that in the colorectal cancer tissues suggesting that it facilitates peritoneal carcinomatosis and liver metastasis. Furthermore, miR-99b, KRAS mutations, and Akt are risk factors for the overall survival of colorectal cancer.

## Highlight

• miRNAs are associated with colorectal cancer, peritonitis carcinomatosis, and liver metastasis.
• The miR-99b, miR-135b, and Akt protein levels, which are highly expressed in peritonitis carcinomatosis and liver metastasis tissues in colorectal cancer play a fundamental role in carcinomatosis and metastasis.
• While miR-99b is highest in the primary tumor, its decrease in liver metastasis suggests that miR-99b may have a protective effect against liver metastasis.
• miR-135 in the highest peritonitis carcinomatosis and liver metastasis compared to the main tissue suggests that it progresses to carcinomatosis and metastasis.

## Introduction

Colorectal Cancer (CRC) is the most common type of cancer among gastrointestinal tumors and the third most common cancer type worldwide.^1^ The most common cause of death is organ metastasis, primarily in the liver, peritoneum, and lungs.^2^ Approximately 60% of patients diagnosed with advanced CRC develop distant metastases within 5 years.^3^ Liver Metastasis (LVM) accounts for approximately one-third of all distant metastasis sites. Another site of distant metastasis is the peritoneum, with approximately one in four patients developing Peritoneal Carcinomatosis (PC).^3^^,^^4^ Although PC may reflect a late stage of the disease, LVM can occur synchronously or metachronously.^4^^,^^5^

Activation of the Epidermal Growth Factor Receptor (EGFR) pathway triggers several downstream signaling pathways, including the RAS/RAF/MEK/Extracellular Signal-Related Kinase (ERK)/Phosphatidylinositol 3-Kinases (PI3K)/AKT, and JAK/STAT3 pathways, to regulate cell growth, survival, and migration in CRC. Deregulated EGFR expression is present in various cancers, including CRC, and increased EGFR expression is present in 25‒77% of CRC.^6^ EGFR activation induces RAS-RAF activation, which leads to the phosphorylation of Mitogen-Activated Protein Kinases (MAPK or MEK), and the activation of ERK.^7^ The MAPK pathway includes KRAS and BRAF, while the RAS/RAF/MAPK pathway regulates cell proliferation, differentiation, apoptosis, and senescence. RAS stimulates this signaling cascade through PI3K. PI3K activation inhibits apoptosis and RAS activation triggers cellular proliferation, thereby promoting cell survival, tumor invasion, and metastasis.^5^, ^6^, ^7^, ^8^ PI3K is associated with cancer because of its role in various cellular processes, including metabolism, inflammation, cell motility, cancer progression, and cell differentiation.^8^ Downstream of PI3K, AKT is activated. The induced activation of Akt can suppress apoptosis in a transcription-independent manner, by phosphorylating and inactivating components of the apoptotic machinery. Mutations related to PI3K/AKT/mammalian Target of Rapamycin (mTOR) have been identified in 29% of all tumors.^9^

MicroRNA (miR, miRNA) is non-coding RNA that is responsible for the modulation of more than half of protein-coding gene expression.^10^^,^^11^ The miR-99 family, which consists of miR-99a, miR-99b, miR-100-5p, and miR-100-3p, plays a vital role in the regulation of a broad range of targets. This includes the mRNAs of tumor suppressors and promoters.^12^ They can act as tumor suppressors in CRC, gastric cancer, prostate cancer, and squamous cell carcinoma, whereas other onco-miRNAs may play roles in hepatocellular cancer and leukemia.^12^, ^13^, ^14^

The miR-135 precursor is a small noncoding RNA involved in the regulation of gene expression.^15^ Nagel et al.^16^ first reported that miR-135a expression is significantly upregulated in colorectal adenomas and carcinomas. Furthermore, the upregulation of several groups of miR-135b-5p has been observed to promote tumorigenic metastasis in other types of cancer, including lung cancer, and head and neck squamous cell carcinoma.^17^^,^^18^ Li et al.^13^ reported that miR-135b-5p may be a promising non-invasive biomarker for diagnosing CRC patients with TNM stage III/IV, and a potential candidate for developing an intervention strategy for CRC.

MiR-135b affects many molecular biological pathways, including homeostasis, osteoporosis, neurological diseases, CRC, and gastric cancer.^16^, ^17^, ^18^, ^19^ In CRC, miRNA-135b can target the 3’-UTR of Adenomatous Polyposis Coli (APC) and restrain its expression, resulting in the aberrant activation of the Wnt signaling pathway.^16^ Furthermore, miR-135b acts as a basic downstream effector of oncogenic pathways, resulting in CRC progression.^20^ Heublein et al.^21^ used the miRNA profiling of primary tumor tissues to identify miRNAs potentially associated with synchronous or metachronous metastatic spread in CRC. In conclusion, the aforementioned literature review found that miRNA expression in CRC primary tumor tissues can predict the location of synchronous or metachronous metastatic spread.^21^

The present study aimed to analyze miR-99b and miR-135b in PC and LVM tissues due to CRC, examine intracellular signaling pathways such as KRAS and Akt genes and investigate their effects on survival.

## Methods

### Patients and ethics

This study enrolled 74 patients who underwent surgery for CRC-associated PC and LVM in general surgery and oncology clinics. The patients were divided into two groups: those who developed PC due to CRC (*n* = 46) (Group 1) and those who developed LVM (diagnosed in preoperative imaging) (*n* = 28) (Group 2). However, 137 paraffin blocks from patients were included in the study. Specimens from patients were analyzed for the AKT gene, KRAS mutation status, miR-99b, and miR-135b. This study was approved by the ethics committee of our hospital (E.71522473-050.01.04-128389-139).

### Inclusion and exclusion criteria

Patients with PC or LVM who underwent surgical treatment between 2012 and 2020 in Sakarya, Türkiye, who were over 18 years of age, and who had paraffin blocks were enrolled in the study. The exclusion criteria were those who did not voluntarily consent to participate, those under the age of 18 years, those who refused to participate in the study, and those with peritoneal cancer due to any other cancer type.

### Histopathological specimens and formation of groups

From our hospital's pathology department archives, 137 slides with PC and LVM due to CRC, including a control group, were evaluated using light microscopy. After examination, the paraffin tissue blocks from the PC, LVM, and control groups were included in the study. Distal and proximal surgical margins were evaluated in the control group. These preparations were evaluated by an experienced pathologist and those suitable for Akt immunostaining were determined. In this study, four groups were created based on the histopathological evaluation of the sections of the 137 samples. These included a CRC (*n* = 49), PC (*n* = 28), LVM (*n* = 35), and control or normal tissue group (*n* = 28).

### Immunohistochemical staining

An ab182729 Akt1-Akt2 (EPR17062) antibody was used for IHC staining. Antibody incubation was performed with the ab182729 Akt1-Akt2 (EPR17062) (Abcam Cambridge CB2 0AX, UK) antibody at a 1:1200 dilution for 60 min. ROCHE/Cell Conditioning 1 (CC1) (EDTA) and CC1 60-min standard protocols were used for antigen retrieval.

A standard UltraView IHC protocol was applied using a ROCHE/UltraView Universal DAB Detection Kit for secondary imaging, and all procedures were performed using a ROCHE/Ventana BenchMark XT IHC Stainer.

### Immunohistochemical scoring system and severity percentage

Akt staining scoring of tumor cells was performed using a scale consisting of five groups. The results were as follows: Grade 0, no staining or staining in less than 10% of the tumor cells; Grade 1: 10‒25%, Grade 2: 26‒50%, Grade 3: 51‒75%, and Grade 4: 76‒100%. IHC was performed using cytoplasmic and nuclear staining.^22^

### Mutation status and detection

KRAS and NRAS mutations were previously detected using the service procurement method, and the data for KRAS and NRAS were obtained from the hospital database. DNA was extracted from histologically confirmed paraffin block samples using the QIAamp DNA FFPE tissue kit (Qiagen, Hilden, Germany). Additionally, the DNA concentrations were measured using the Qubit dsDNA HS assay kit (Qubit 4.0 Fluorometer, ThermoFisher Scientific Inc., Massachusetts, USA). DNA obtained was then amplified by nested PCR (2-step) using primers specific to codons in which mutations were observed in exons 1, 2, and 3rd exons of KRAS. These products were labeled using fluorescently labeled ddNTPs so that the target mutation sites could be detected using minisequencing.^23^ KRAS and NRAS mutation analyses were performed using capillary electrophoresis.

### RT-PCR analysis

#### Deparaffinization-RNA isolation

Paraffin blocks of the primary tumor, PC, LVM, and normal tissues collected between 2016 and 2020 from patients, identified by RNA isolation were used. For RNA isolation from paraffin blocks, 5‒10 sections of 20 µm thickness were taken from paraffin-embedded tissues with a microtome device and placed in tubes. To remove the paraffin from these sections 1 mL of xylene was added to each tube and vortexed. The vortexed samples were centrifuged at 12.000 rpm for 5 min (Nüve, Turkey). After centrifugation, the supernatant was discarded, and the cells were washed twice with pure alcohol. The washed samples were then dried for 15 min at room temperature. Two hundred microliters of buffer solution containing proteinase K were added to each of the dried samples and vortexed for 5 min. Each sample containing the buffer was incubated overnight in a heated block, at 55°C to melt the paraffin. The following day, the samples were incubated at 90°C for 30 min. After incubation, the samples were used for RNA isolation.

Paraffin-free tissue samples obtained from paraffin blocks were subjected to RNA isolation using TRIzol (Thermo Fisher Scientific) RNA isolation reagent. For this purpose, 250 µL TRIzol was added to each sample taken from the heating block and vortexed. After centrifugation, the upper three phases obtained from each sample were transferred to a clean tube. Then, 500 µL of isopropanol was added to each sample of the obtained supernatant and incubated at room temperature. After centrifugation, 1 mL of 75% ethanol was added to the pellet obtained, by removing the supernatant from each sample and centrifuging at 7500× g for 5 min. The dried samples were added to 30 µL of distilled water and incubated at 55°C in a heating block, and then stored at -80°C.

#### cDNA synthesis

A Qubit 4 (Thermo Scientific) spectrophotometer was used to quantify the total RNA obtained from each sample before cDNA conversion. cDNA synthesis was performed using the quantified RNAs with the miRNA All-In-One cDNA Synthesis Kit (Abm, Canada). The obtained cDNAs were stored at -20°C for use in RT-PCR analysis.

### Reverse transcription polymerase chain reaction (RT‒PCR) analysis

To analyze changes in the expression levels of miR-99b-5p (MPH03979, Abm, Canada) and miR-135b-5p (MPH02185, Abm, Canada) from the obtained cDNAs, RT-PCR was performed using Step One Plus Real-Time PCR Systems (v. 2.0, Applied Biosystems) with Luna® Universal qPCR Master Mix (NEB, UK) in 96-well optical reaction plates. U6-2 (MPH00001, Abm, Canada) was used as a control miRNA to normalize the data.^24^

### Statistical analysis

Statistical analyses were performed using SPSS (version 22.0; IBM Corp., Armonk, NY, USA). The numbers of patients and percentages (%) were used as categorical data. Categorical variables were tested using a continuity-corrected χ^2^ test and Fisher's exact test to compare qualitative data. The normality of the data distribution was determined using the Shapiro-Wilk test. In the analysis, it is shown as the mean value or median (25th percentile‒75th percentile) according to the normal distribution. Student's *t*-test or Mann-Whitney *U* test was used according to a normal distribution in pairwise, independent groups. The data obtained from RT-PCR analysis were evaluated using software (https://dataanalysis.qiagen.com/pcr/arrayanalysis.php). Increases or decreases in miRNA expression levels were determined as fold-change using the 2^−ΔΔCT^ method. To determine the best cut-off points for quantitative miR-99, miR-135b, and Akt measurements in predicting overall survival, we used the web-based Cutoff Finder algorithm (http://molpath.charite.de/cutoff) written in the R program. The best cutoff for these variables was used. The univariate effects of all possible variables considered to have an impact on overall survival were analyzed using Cox proportional hazards regression models. The factor(s) that were the most significant determinants of OS was then investigated by constructing proportional hazard models using multivariate Cox regression analysis. Whether there was a statistically significant difference between miR-99, miR-135b, and Akt levels and the locations where miR-99b, miR-135b, and Akt levels were measured was investigated using Friedman's test. If the statistical results of the Friedman test were significant, the situation(s) causing the differences were determined using the Dunn-Bonferroni multiple comparison test. The Spearman's ordinal number correlation test was used to determine whether there was a statistically significant correlation between continuous numerical variables. Results were considered statistically significant if *p* < 0.05.

## Results

### Clinical features

A total of 74 patients participated in the study: 29 females (39.2%) and 45 males (60.8%). The median age was 58.5 (47.5‒68) years, with 33.7% being over 65 years. CRC was located in the colon (55.4%) and rectum (44.5%). In addition, most cancers were located on the left side (77% left vs. 23% right). The recurrence rate in previously operated patients was 47.9%. The number of patients who received neoadjuvant chemotherapy was 17 (23.6%). Collectively, the rate of PC was 62.1%, and the rate of LVM was 37.8%. An extensive Glisson capsule metastasis in operation (9.4%) was found in the PC group but was not classified to be a form of clinical distant metastasis. The mean age of the patients with PC was lower than that of the group with LVM (58.1 vs. 65.6 years) (*p* = 0.002). Additionally, the number of recurrent cases was higher in the PC group (38.4% vs. 9.6%) (*p* = 0.004). However, the tumor stage was lower in the PC-developing group in this study (*p* = 0.017) (Table 1).

**Table 1:** Assessment of the clinical data between groups.

	Total (*n* = 74)	Peritoneal carcinomatosis (*n* = 46) (Group 1)	Liver metastasis (*n* = 28) (Group 2)	*p*-value
Age (year)	59.917 (13.500)	58.1 (12.145)	65.6 (18.610)	0.002^c^
Gender, n (%)				
Female	29 (39.2)	19 (25.7)	10 (13.5)	0.578^d^
Male	45 (60.8)	27 (36.5)	18 (13.5)	
ASA	3 (2.5 ‒ 3)	3 (3‒3)	3 (2‒3)	0.382^a^
Body Mass Index	24.3 (5.283)	22.8 (4.931)	29 (3.605)	0.06^c^
Localization, n (%)				0.332^d^
Colon	41 (55.4)	28 (37.8)	13 (17.6)	
Rectum	33 (44.6)	18 (24.3)	15 (20.3)	
Side, n (%)				0.595^d^
Left	57 (77)	34 (45.9)	23 (31.1)	
Right	17 (23)	12 (16.2)	5 (6.8)	
KRAS status, n %)				0.968^d^
Wild type	32 (62.7)	17 (33.3)	15 (29.4)	
Mutant type	19 (37.3)	11 (21.6)	8 (15.7)	
NRAS positivity	2 (2.7)	1 (2.1)	1 (3.5)	>0.999^b^
Tumor diameter	5.2 (1.699)	5.67 (1.714)	4 (1)	0.134^c^
Recurrent case, n (%)	35 (47.9)	28 (38.4)	7 (9.6)	0.004^d^
Neoadjuvant treatment, n (%)	17 (23.6)	9 (12.5)	8 (11.1)	0.613^d^
CEA	11.4 (5.9‒93)	12 (4‒97)	10.8 (9.65‒17.4)	0.152^a^
CA19-9	49.4 (7‒262)	70 (18‒256)	2 (2‒1645)	0.096^a^
Adjuvant treatment, n (%)	65 (94.2)	40 (58)	25 (36.2)	>0.999^b^
Radiotherapy, n (%)	24 (35.8)	12 (17.9)	12 (17.9)	0.253^a^
Staging	4 (4‒4)	4 (3‒4)	4 (4‒4)	0.017^a^
Length of hospital	17.5 (15.5‒37.5)	18 (15‒35)	17 (16.5‒53)	0.014^a^
Follow-up	17.2 (13.7‒27.2)	15.6 (13.1‒20.4)	31.3 (22.9‒41.7)	<0.001^a^
Lymph node invasion, n (%)	51 (76.1)	34 (50.7)	17 (25.4)	0.075^d^
Mucinous component, n (%)	25 (39.1)	22 (34.4)	3 (4.7)	0.001^d^
Grade (median)	2 (1.5‒2)	2 (2‒2)	2 (1‒2)	0.306^a^
Lymphovascular invasion, n (%)	38 (70.4)	21 (38.4)	17 (31.5)	0.635^d^
Neural invasion, n (%)	29 (67.4)	17 (39.5)	12 (27.9)	0.279^d^

ASA, American Society of Anesthesiologists; CA19-9, Carbohydrate Antigen 19-9; CEA, Carcinoembryonic Antigen.

Definitive statistics: median (25 percentage‒75 percentage) or percentage (%) or mean (standard deviation).

a Mann Whitney *U* test.

b Fisher's exact test.

c Student's *t*-test.

d Continuity corrected χ^2^ test.

### Histopathological examination

The median stage was 4 (3‒4) (78.4%) according to the TNM staging system for colorectal cancer 8th ed., 2017 of the American Joint Committee on Cancer (AJCC). Histopathological examination (*n* = 74) revealed that Grades 1, 2, and 3 were present in 29.8%, 55.3%, and 14.6%, respectively. The mucinous component, lymphovascular involvement, and neural involvement rates were 39.1%, 70.4%, and 67.4%, respectively. The median number of surgically removed LNs was 16 (12‒25). However, the rate of lymph node positivity was only 76.1% (Table 1).

**Fig. 1:**
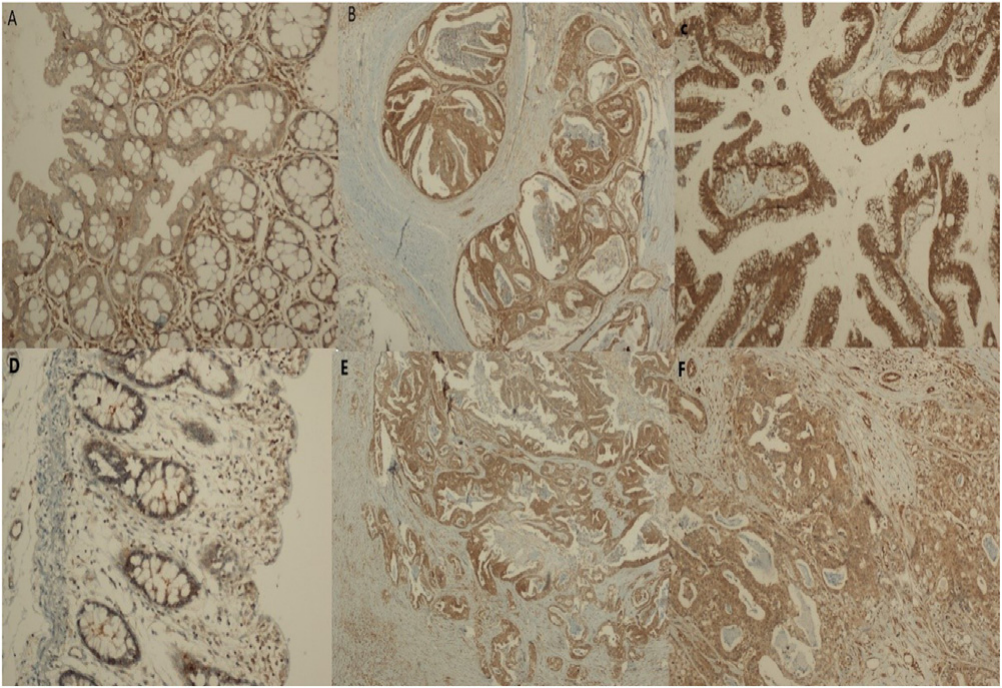
(a) Weak cytoplasmic Akt-staining in a normal colon mucosa (×100, IHC). (b and c) Intense, diffuse immunopositivity for Akt in the colon adenocarcinoma (×100 and ×200, respectively). (d) Weak cytoplasmic Akt-staining in the normal colon mucosa (×200, IHC). (e) Intense and diffuse cytoplasmic Akt-staining in the peritoneal carcinomatosis (×100, IHC). (f) Cytoplasmic Akt-staining in the liver metastasis (×200).

**Fig. 2:**
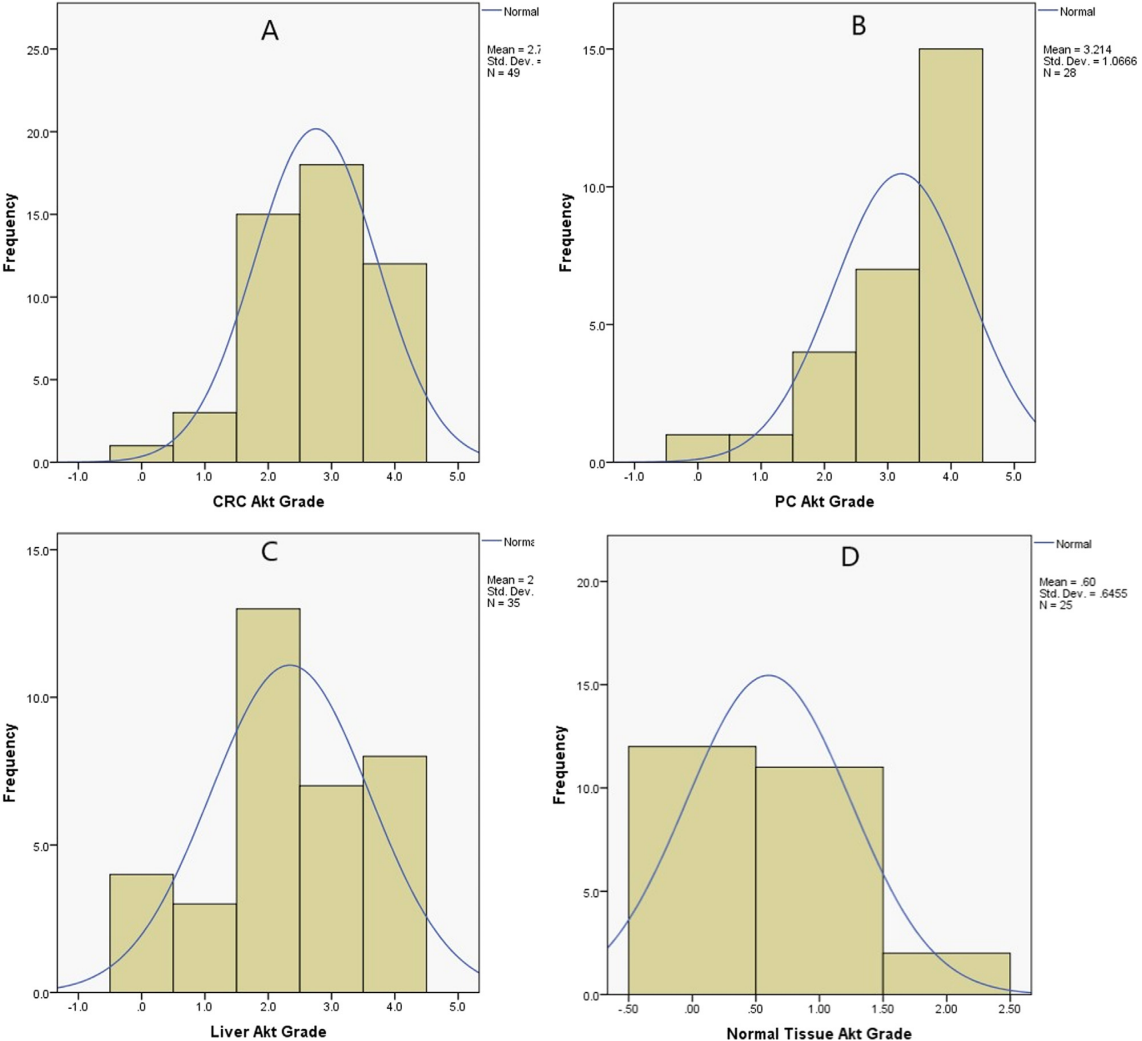
Immunohistochemical staining grade histograms of Akt in colorectal cancer (A), peritoneal carcinomatosis (B), liver metastasis (C), and normal tissue (D). CRC, Colorectal Cancer; LVM, Liver Metastasis; PC, Peritoneal Carcinomatosis.

The IHC grade scoring system for the Akt gene showed that the median grade was 3 (2‒3) for CRC, 4 (3.5‒4) for PC, 4 (3.5‒4) for LVM, and 1 (1‒1) for normal tissue (*p* < 0.001). The post hoc analysis showed that the Akt gene grade of the scoring system in CRC, PC, and LVM was higher than that in normal tissues (*p* < 0.001) (Tables 1 and 2) (Fig. 1, Fig. 2).

**Table 2:** Comparison of different Akt tissues immunohistochemical scoring system and severity (%).

	CRC (*n* = 49) (%)	PC (*n* = 28) (%)	LVM (*n* = 35)⁎⁎ (%)	Normal Tissue (*n* = 25) (%)	*p*-value†
AKT grading					<0.001
0	1 (2)^a^	1 (3.6)^b^	4 (11.4)^c^	12 (48)^a^^,^^b^^,^^c^	
1	3 (6.1)^a^	1 (3.6)^b^	3 (8.6)^c^	11 (44)^a^^,^^b^^,^^c^	
2	15 (30.6)^a^	4 (14.3)^b^	13 (20)^c^	2 (8)^a^^,^^b^^,^^c^	
3	18 (36.7)^a^	7 (25)^b^	7 (22.9)^c^		
4	12 (24.5)^a^	15 (53.6)^b^	8 (22.9)^c^		
Akt* (%)	70.0 (42.5‒80.0)^d^	80.0 (62.5‒80.0)^e^	40.0 (30.0‒77.5)^f^	5.0 (5.0‒15.0)^d^^,^^e^^,^^f^	<0.001

CRC, Colorectal Cancer; LVM, Liver Metastasis; PC, Peritoneal Carcinomatosis.

⁎ Akt severity percentage (%).

⁎⁎ Patients with Group 2 and an intensive Glisson capsule metastasis of Group 1. Definitive statistics; median (25 percentage‒75 percentage) or percentage (%).

† Friedman test.

a CRC vs. Normal tissue Akt (*p* = 0.001).

b PC vs. Normal tissue Akt (*p* < 0.001).

c LVM vs. Normal tissue Akt percentage (*p* = 0.026).

d CRC vs. Normal tissue Akt (%) (*p* < 0.01).

e PC vs. Normal tissue Akt percentage (*p* < 0.01).

f LVM vs. Normal tissue Akt) (*p* < 0.01).

### Assessment of KRAS and NRAS

In the present study, the KRAS and NRAS mutation rates were 37.3% and 2.7%, respectively. Mutations in the PC and LVM groups were 21.6% and 15.7%, respectively (*p* = 0.968). In the evaluation of patients with KRAS mutations in terms of tumor stage, the median stage of the wild-type patients was 4 (3‒4), and the median stage of the mutant-type patients was 4 (4‒4) (*p* = 0.601). In addition, when KRAS mutation status, age, tumor localization, body mass index, tumor size, CEA, CA19-9 levels, and PCI were compared, no statistically significant differences were found (*p* > 0.05). In contrast, when KRAS status and the side of the tumor were compared, wild-type CRCs had left-sided localization (52.4%), whereas mutant-type CRCs had right-sided localization (21.4%) (*p* = 0.014) (Table 1).

### Determination of alteration in miRNA expression

MiR-99b and miR-135b expression levels were normalized to U6-2 control miRNA, and the results were evaluated in the main tumor, PC, LVM, and normal tissues of patients with CRC.

**Fig. 3:**
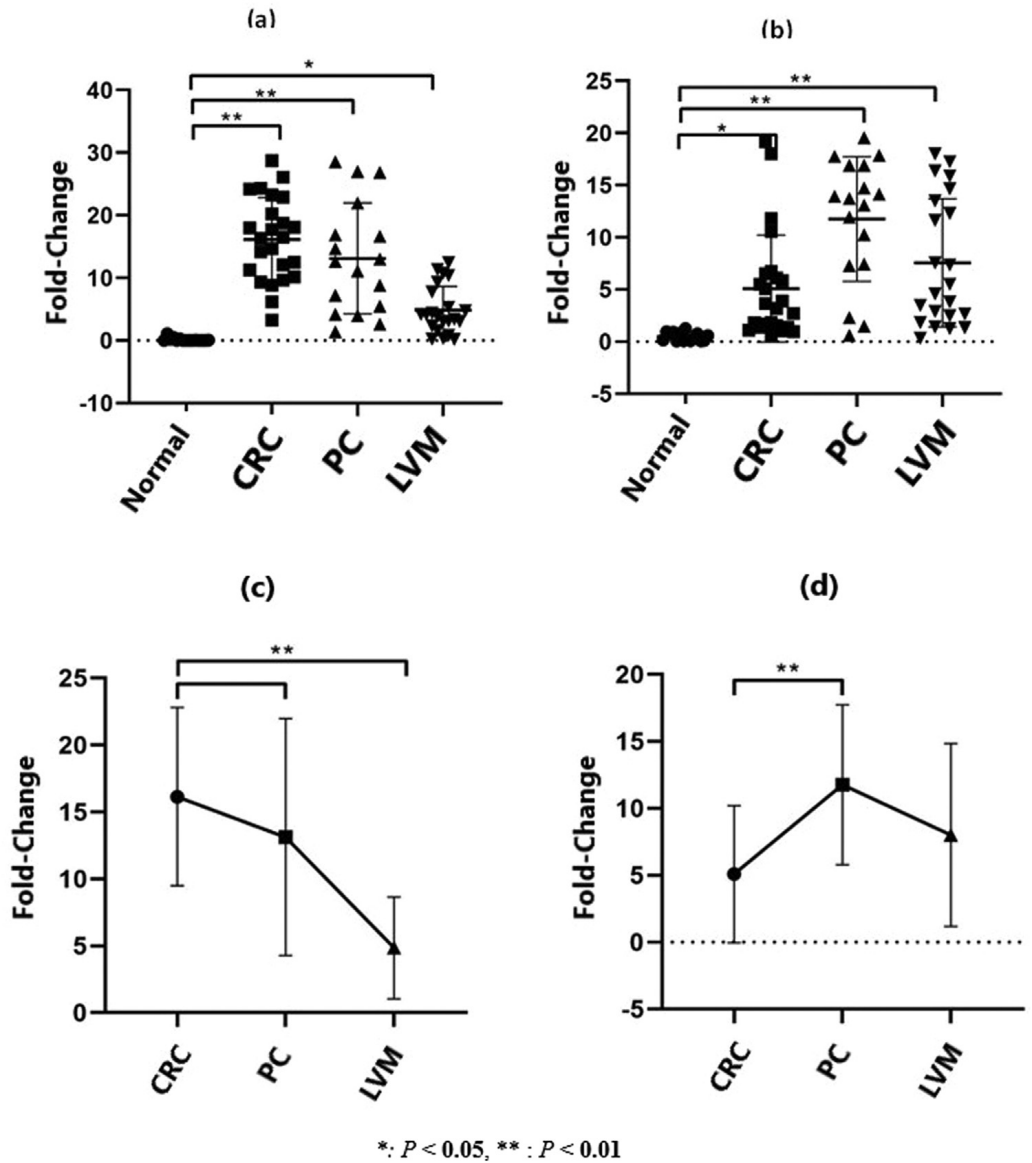
Determination of (a) miR-99b and (b) miR-135b expression levels changes in the tumor, PC, and LVM tissues compared with normal tissue (*p* < 0.05*; *p* < 0.01**). CRC, Colorectal Cancer; LVM, Liver Metastasis; PC, Peritoneal Carcinomatosis.

According to the results of RT‒PCR analysis, when the expression levels of miR-99b and miR-135b in CRC tumor tissues were compared with those in normal tissues, there was a statistically significant increase of 19.7-fold (*p* = 0.001) and 3.53-fold (*p* = 0.027), respectively. In addition, compared with those in normal tissues, the expression levels of miR-99b and miR-135b in PC tissues were increased by 12.55-fold (*p* = 0.001) and 9.68-fold (*p* = 0.001), respectively. Whereas in LVM tissues, the expression levels were increased by 3.97-fold. (*p* = 0.038) and 5.23-fold (*p* = 0.002), respectively (Fig. 3) (Table 3). Although the expression level of miR-99b increased to the highest level in tumor tissue compared with that in normal tissue, the expression level of miR-135b was the lowest (Table 3) (Fig. 3).

**Table 3:** Fold changes and p values in miR-99b and miR-135b expression levels in the tumor, peritoneal metastasis and liver metastasis tissues compared to normal tissue (A). Fold changes and p-values in miR-99 and miR-135b expression levels in peritoneal metastasis and liver metastasis tissues compared to tumor tissue (B).

A	Tumor		Peritoneal carcinomatosis		Liver metastasis	
	Fold-change	p	Fold-change	p	Fold-change	p
Hsa-miR-99b	19.7	0.001	12.55	0.001	3.97	0.038
Hsa-miR-135b	3.53	0.027	9.68	0.001	5.23	0.002

			Peritoneal carcinomatosis		Liver metastasis	
B			Fold-change	p	Fold-change	p

Hsa-miR-99			0.64	0.145	0.2	0.001
Hsa-miR-135b			2.74	0.001	1.48	0.116

In addition, compared with tumor tissues in PC and LVM, miR-99b expression levels were found to be 0.64- (*p* = 0.145) and 0.20-fold (*p* = 0.001) higher, respectively. However, the expression levels of miR-135b were increased by 2.74-fold (*p* = 0.001) and 1.48-fold (*p* = 0.116), respectively. No statistically significant differences were found in miR-99b, miR-135b, and Akt protein levels in CRC, PC, LVM, and normal tissues between wild-type and mutant KRAS (*p* > 0.05) (Table 3) (Fig. 3).

### Risk factors affecting overall survival

Kaplan-Meier survival analysis showed that the survival rate was 24.3%, and the 1‒3 and 5-year overall survival rates were 89.2%‒36.4% and 22.0%, respectively. On the other hand, the mean life expectancy was 40.0 (95% CI 31.6‒48.4) months.

**Fig. 4:**
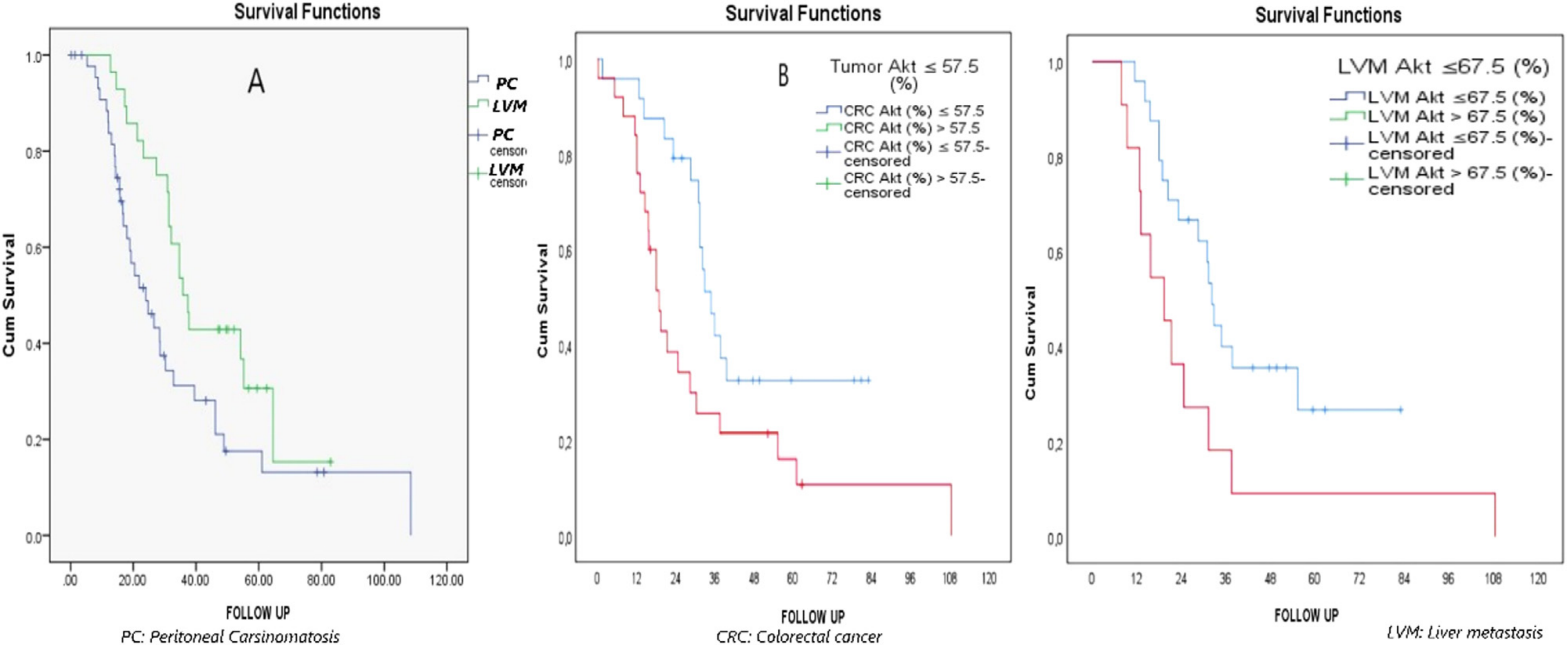
Overall survival analysis in Kaplan Meier Curve in groups.

Furthermore, Kaplan-Meier survival analysis revealed that the median survival of PC cases was lower than that of LVM cases (24 vs. 35.800 months) (*p* = 0.027) (Fig. 4). The mortality rate in patients with primary tumor Akt > 57.5 compared with those with primary tumor Akt ≤ 57.5 was statistically significant, at 2.178 times (95% CI 1.113‒4.261) (*p* = 0.023). Similarly, the mortality rate was significantly higher, by 2.322 times (95% CI 1.045‒5.160) in patients with LVM Akt > 67.5 than in those with LVM Akt ≤ 67.5 (*p* = 0.039) (Fig. 4).

The best cutoff points were obtained to determine the best cutoff points for quantitative miR-99b and miR-135b levels, and Akt protein for predicting overall survival. All variables found to be *p* < 0.25 in univariate statistical analyses were included in the regression model as candidate factors. The tumor Akt level, neural invasion, and KRAS mutation status were the most important determinants for overall survival. Regardless of other factors, the mortality rate continued to increase statistically, up to 4,591 times (95% CI 1.527‒13,801) in those with main tumor Akt > 57.5 compared with those with main tumor Akt ≤ 57.5 (PS = 0.007). When adjusted for other factors, the presence of neural invasion (HR = 6.264; 95% CI 1.482‒26.470 and *p* = 0.013) and mutant KRAS (HR = 2.757; 95% CI 1.039‒7.314 and *p* = 0.042) increased the mortality rate (Table 4).

**Table 4:** Most predictive factors on overall survival – multivariate Cox's proportional hazards regression model.

	Hazard ratio	95% Confidential Interval		Wald	*p*-value
		Lower bound	Upper bound		
Model 1					
Localization (Rectum)	1.068	0.384	2.970	0.016	0.899
T-staging	2.491	0.828	7.489	2.639	0.104
Lympho-vascular invasion	1.951	0.692	5.505	1.597	0.206
Neural invasion	6.264	1.482	26.470	6.227	0.013
CRC Akt (%) (> 57.5)	4.591	1.527	13.801	7.367	0.007
KRAS mutation	2.757	1.039	7.314	4.148	0.042
Model 2					
Age	1.008	0.861	1.179	0.009	0.925
Staging	0.070	0.004	1.298	3.188	0.074
Recurrent case	147.100	2.951	7332.552	6.263	0.012
Lymph node positivity	9.552	0.616	148.239	2.602	0.107
Neural invasion	92.033	0.537	15762.401	2.970	0.085
CRC Akt (%) (> 57.5)	1.096	1.026	1.170	7.492	0.006
miR-99b-CRC	1.188	1.004	1.406	4.008	0.045
KRAS mutation	12.734	0.361	449.481	1.958	0.162

Akt, Akt gene; CRC, Colorectal Cancer; T, Tumor deep.

In line with clinical predictions, the factors that determined overall survival in the current model were primary tumor Akt level, presence of recurrence, and primary tumor miR-99b level. Independent of other factors, as the tumor Akt level increased (HR = 1.096; 95% CI 1.026‒1.170 and *p* = 0.006), recurrence developed (HR = 147.100; 95% CI 2.951‒7332.552 and *p* = 0.012), and the primary tumor miR-99b expression level increased (HR = 1,188; 95% CI 1.004‒1.406 and *p* = 0.045). Furthermore, the death rate increased (Table 4).

## Discussion

CRC is the third most common cancer and the third most common cause of cancer-related deaths in the United States.^1^ The annual incidence was 36.5/100000 individuals between 2014 and 2019.^1^,^2^ The incidence is similar regardless of sex and increases significantly with age. The median age at diagnosis is approximately 70 years in developed countries.^1^,^2^

Despite its strong hereditary components, most cases of CRC are sporadic and develop slowly over several years along the adenoma-carcinoma sequence. The cornerstones of treatment include surgery, neoadjuvant radiotherapy (for patients with rectal cancer), and adjuvant chemotherapy (for patients with stage III/IV and high-risk stage II colon cancer). The 5-year relative survival rate is > 90% in patients with stage I disease and > 10% in patients with stage IV disease (3‒5.29).

MiRNAs represent the most studied class of non-coding RNAs and are responsible for the negative modulation of up to 60% of protein-coding gene expression.^10^,^11^ These miRNAs bind to target mRNAs that are complementary to their nucleotide sequences and regulate post-transcriptional gene expression via translational repression or mRNA degradation. In recent studies, altered miRNA expression has been investigated in cancer cells in which uncontrolled cell division occurs. During the initiation and progression of cancer, miRNAs function as tumor suppressors or oncogenes, depending on the characteristics of the target gene.^11^,^12^

The initial association of miRNAs with CRC by Michael et al.^23^ began 15 years ago, when they reported decreased expression of miR-143 and miR-145 in colon cancer tissues compared with normal colon tissues. Since then, several studies have investigated the role of miRNAs in CRC.^11^,^12^,^25^ Given the evolutionary progression of CRC from adenoma, an in-depth understanding of miRNA-modulated molecular mechanisms has become a major challenge.^11^ Alterations in the Wnt/β-catenin, EGFR, TGFβ, and TP53 signaling pathways have been shown to affect the survival, proliferation, invasion, and metastasis of CRC.^11^,^26^ Therefore, many studies have focused on these pathways to establish miRNA-mRNA interaction networks and complete the molecular puzzle underlying CRC development, progression, and metastasis. The EGFR signaling pathway plays a critical role in normal embryogenesis, as well as in cellular proliferation, differentiation, migration, and apoptosis. Genetic changes in EGFR may lead to the development of cancer. Approximately 50% of CRC cases exhibit EGFR amplification and mutational activation of KRAS and BRAF downstream mediators.^11^,^22^

The microRNA-99 family is evolutionarily conserved, and its behavior may vary according to the miRNAs they target in different diseases. They are typically oncogenic in leukemia, but their functional roles in different cancers alternate between those of tumor suppressors and tumor promoters. Thus, this study demonstrates the dual role of this miR family as onco-miRs and tumor suppressor miRs in different cellular contexts. In addition, the miR-99 family may be involved in the modulation of macrophage inflammatory responses and the biology of T-cell subsets. Therefore, it plays critical role in maintaining tissue homeostasis, establishing peripheral tolerance, and resolving inflammatory reactions.^11^,^12^

The miR-99 family plays an essential role in CRC. MiR-99 family members, acting as TSmiRs, can generally prevent cancer metastasis^14^,^15^,^17^ In a study of 56 primary CRC clinical samples and related LVMs, Li et al.^13^ showed that miR-99b-5p downregulation in CRC was a common event during LVM. MiR-99b-5p overexpression inhibits mTOR, indicating that miR-99b-5p blocks CRC metastasis by targeting Mtor.^17^

The miR-135 miRNA precursor is a small non-coding RNA involved in the regulation of gene expression.^16^,^27^ MiR-135b-5p is a conserved transcript among mammals and is located at the 1q32.1, gene locus in humans. In early 2008, Nagel et al.^16^ reported that miR-135a expression was significantly upregulated in colorectal adenomas and carcinomas. Furthermore, the upregulation of several groups of miR-135b-5p has been observed to promote tumorigenic metastasis in other types of cancer, including lung cancer and head and neck squamous cell carcinoma.^17^, ^18^, ^19^ Increasing evidence has shown that miR-135b-5p is involved in CRC transformation and progression.^20^,^21^ However, the precise mechanism by which miR-135b-5p is involved in CRC metastasis is complex and unclear. Li et al.^28^ reported that miR-135b-5p may be a promising non-invasive biomarker for diagnosing CRC patients with TNM stage III/IV and a potential candidate for developing an intervention strategy for CRC.

In our study, patients with advanced colon and rectal cancer diagnosed histopathologically as having colorectal adenocarcinoma were divided into two groups: patients with LVM and those with PC. We investigated the survival effect through changes in Akt protein levels, KRAS mutation status, and miR-99b and miR-135 expression levels in paraffin blocks of LVM and PC.

In this study, the Akt gene, IHC grade classification score, and density percentage in tissues with CRC, PC, and LVM were significantly higher than those in normal tissues (*p* < 0.001). According to the current evaluations, a percentage of Akt level in the main tumor of over 57.5% significantly increases the death rate (*p* = 0.023). Similarly, in patients with LVM, if the percentage of Akt was > 67.5%, the mortality rate increased significantly (*p* = 0.039). These findings suggest that Akt in the main tumor increases CRC formation and promotes metastasis to the liver.

MiR-99b and miR-135b expression levels in primary CRC tissues were significantly higher than those in normal tissues (*p* = 0.001 and *p* = 0.027, respectively). In addition, miR-99b and miR-135b expression levels were significantly higher in PC and LVM tissues than in normal tissues (*p* < 0.05). The most important determinants for overall survival were Akt, neural invasion, and KRAS mutation status. Regardless of other factors, the mortality rate continued to increase statistically, to 4,591 times in patients with primary tumor Akt > 57.5 compared with those with CRC tumor Akt ≤ 57.5 (*p* = 0.007). When adjusted for other factors, the presence of neural invasion (*p* = 0.013) and KRAS mutations (*p* = 0.042) increased the mortality rate. In line with clinical predictions, the factors that most determined overall survival in the current model were Akt, the presence of recurrence, and miR-99b-CRC. Independent of other factors, the death rate increased as Akt levels (*p* = 0.006) and miR-99b expression levels increased in the main tumor (*p* = 0.045), and as recurrence occurred (*p* = 0.012).

Although the expression level of miR-99b was the highest in the main tissue, the expression level of miR-135b was the lowest. In addition, although the expression level of miR-99b was decreased in PC and LMV compared with that in primary tumor tissue, the expression level of miR-135b was higher than that in CRC tissue. The reduction in miR-99b expression in PC and LVM tissues compared with that in CRC tissues suggests that it has a protective effect on invasion and metastasis. In contrast, the increased expression of miR-135b in PC and LVM compared to that in the parent tissue suggests that it increases invasion and metastasis. We believe that due to the small sample size in our study, there was no correlation between Akt levels, KRAS mutations, and miR-99b and miR-135b expression levels. Therefore, further experiments should be performed to evaluate the association between the PI3K/Akt/mTOR signaling pathway and changes in miR-99b and miR-135b expression levels.

Although the results of the current study are similar to those reported in the literature, they reveal important new information. The following studies support our results: Li et al.^13^ showed that miR-99b is down-regulated in the liver metastasis of CRC; Fujino et al.^14^ reported that downregulation of microRNA-100/microRNA-125b is related to lymph node metastasis in early CRC; and Zheng et al.^15^ reported that the long noncoding RNA HAGLROS adjusts apoptosis and autophagy in CRC cells by sponging miR-100 to target ATG5 expression. In contrast, the upregulation and pro-oncogenic behavior of miR-135 in advanced stages is similar to our results.^27^,^28^ In addition to the aforementioned studies, our study is the first to reveal the behavioral characteristics of miR-99b and miR-135b in PC caused by CRC.

Our study has some limitations. First, the most important limiting factor of our study was its small sample size. Second, although KRAS mutations were detected in most patients, they were not detected in all patients. Additionally, phospho-Akt levels were not measured separately by IHC, to better understand the role of the PI3K/Akt/mTOR signaling pathway in CRC progression.

## Conclusion

The high levels of miR-99b and miR-135b, and increased Akt protein, which are highly expressed in PC and LVM tissues in CRC, were significantly different from those in normal tissues. This indicates that they play an important role in PC and LVM. While miR-99b expression was highest in the primary tumor, its decrease in LVM suggests that miR-99b may have a protective effect against liver metastasis. In contrast, the detection of miR-135 in the PC and LVM compared with the main tissue suggests that it promotes progression to PC and LVM. Moreover, MiR-99b, KRAS mutations, and Akt are risk factors for poor overall survival in CRC.

## Conflicts of interest

The authors declare no conflicts of interest.
